# Bayesian analysis supports the role of neoadjuvant chemoradiation followed by surgery for resectable locoregional esophageal cancer

**DOI:** 10.1111/1759-7714.14619

**Published:** 2022-08-21

**Authors:** Alessandro Rizzo

**Affiliations:** ^1^ Struttura Semplice Dipartimentale di Oncologia Medica per la Presa in Carico Globale del Paziente Oncologico "Don Tonino Bello", I.R.C.C.S. Istituto Tumori "Giovanni Paolo II" Bari Italy

To the Editor

The optimal perioperative treatment for resectable locoregional esophageal cancer is uncertain and several questions remain unanswered in this setting. In the current systematic review and network meta‐analysis (NMA) published by Bao et al.,^1^ the authors pulled together 20 clinical trials for a total of 4384 patients with resectable locoregional esophageal cancer.^1^ According to the results of the Bayesian NMA, Bao and colleagues^1^ suggested that neoadjuvant chemoradiation followed by surgery (NCRT + S) may be the most efficient neoadjuvant treatment for resectable locoregional esophageal cancer. At the same time, the authors highlight that NCRT + S may increase the risk of postoperative mortality, but not morbidity.^1^

Bao et al.^1^ performed Bayesian NMA to optimize data extrapolation and to compare different treatments when no direct comparative trial was available and to obtain more precise effect estimated by jointly considering direct and indirect comparisons.^2^ Here, the authors used rigorous, well‐accepted methods to assess evidence across clinical trials, also acknowledging important limitations.

However, we believe some methodological issues would deserve discussion.

First, Bayesian NMA—similarly to pairwise meta‐analysis—may be associated with the inflation of false‐positive (type 1) and false‐negative (type 2) errors; because these specific errors have been suggested to play an important role to validating true‐positive as well as true‐negative findings in meta‐analyses, this issue should be carefully considered.^3^

In our view, Bao et al. are to be commended for this interesting NMA aimed at evaluating a timely topic in resectable locoregional esophageal cancer. At the same time, Bayesian NMA has some limitations that should be considered and cannot replace head‐to‐head clinical trials comparison. Based on these premises, the NMA by Bao and colleagues^1^ further emphasizes the need for large‐scale, well‐designed clinical trials aimed at investigating the best pattern of neoadjuvant therapy for resectable locoregional esophageal cancer.

We invite the authors to share their view on these remarks.

## FUNDING

This research received no financial support.

## CONFLICT OF INTEREST STATEMENT

The authors declare that the research was conducted in the absence of any commercial or financial relationships that could be construed as a potential conflict of interest.

## AUTHOR CONTRIBUTION STATEMENT

All authors contributed to the article and approved the submitted version.
